# The impact of social media on risk perceptions during COVID-19 in Saudi Arabia

**DOI:** 10.3389/fpubh.2022.898041

**Published:** 2022-08-03

**Authors:** Khadijah Angawi, Mutlaq Albugmi

**Affiliations:** ^1^Department of Health Services and Hospital Administration, Faculty of Economics and Administration, King Abdulaziz University, Jeddah, Saudi Arabia; ^2^King Saud University Medical City, King Saud University, Riyadh, Saudi Arabia

**Keywords:** COVID-19, risk perception, social media, self-efficacy, Saudi Arabia

## Abstract

**Background:**

Social media is considered a critical source for seeking health information, especially during outbreaks. During the Coronavirus disease 2019 (COVID-19) pandemic, social media played an important role in disseminating information. However, it has been a source of misinformation in many communities throughout the pandemic. Whether this disseminated information has a positive or negative impact, individuals' risk perceptions of disease are influenced. It is important to explore factors that build public behaviors and their adaptation of risk reduction measures during the COVID-19 pandemic. Therefore, the aim of this study is to determine the role of social media and its impact on the risk perceptions of the COVID-19 in Saudi Arabia.

**Methods:**

This was a cross-sectional study, and participants were recruited using different social media outlets between August to October 2020. The survey was delivered through Qualtrics platform targeting Saudi Arabian residents over the age of 18 years old. The questionnaire was delivered in English and Arabic. A convenience sampling was used to recruit participants to the study. The survey link was posted on several social media platforms.

**Results:**

A total of 2,680 respondents completed the online survey. The results showed that male gender, individuals earning 4,000–12,000 SAR, and employed had positive and significant relationships with risk perception compared to their counterparts (β: 0.044, *p*-value: 0.035 and β: 0.051, *p*-value: 0.041, β: 0.108 *p*-value: < 0.001, β: 0.119 *p*-value: < 0.001), respectively. In second block, individuals exposed to social media had higher risk perception (β: 0.096, *p*-value < 0.001). In third block, self-efficacy was significantly but negatively associated with risk perception, indicating individuals who were self-efficient were less likely to perceive risk for the COVID-19 (β: −0.096, *p*-value < 0.001). There was no interactive effect of social media and self-efficacy on risk perception.

**Conclusion:**

The current study results show that social media exposure to the COVID-19 information has a positive impact in shaping an individuals' risk perception. The study also suggests that there is a need for public officials and policymakers to develop effective communication strategies through risk communication campaigns targeted at women, individuals with lower socioeconomic status, and those who are single as they showed a negative relationship with risk perception.

## Introduction

Today's society is facing a major risk in the form of the coronavirus pandemic. In December 2019, the first case of a novel Coronavirus disease 2019 (COVID-19) was identified in Wuhan, China. Soon, thousands of people were affected (1). Despite the growing outbreak, Chinese health officials and the government remained unclear about the origin. Since then, the uncertainty around COVID-19 has contributed to the spread of the virus worldwide, and the number of cases has rapidly been increasing, killing millions of people worldwide (1). To date, various variants of COVID-19 have emerged around the world (2).

Both population growth and change in human behavior have contributed to a rise in outbreaks of emergent infectious diseases (3). Today's widespread uses of technology and internet access have allowed healthcare communication to expand on a global scale. Social media is considered a critical source for health information in some countries, especially during infectious disease outbreaks (3). Social media can be useful in communicating information on emerging infectious diseases and medical information, positively impacting people's perceived risks and decision-making processes (3). Furthermore, social media has an impact on an individual's risk perceptions, which have been moderated by other factors such as self-efficacy (3). Individuals interpret information based on their risk perception of the disease. Furthermore, an individual's risk perceptions influence the protective behaviors in facing an outbreak of an infectious disease (4).

Ideally, governments and public officials are the most trusted sources that play a critical role in disseminating information (5). The public's trust in social media has been known to positively influence their risk perceptions in terms of both overreactions to the epidemic and compliance with self-quarantine (5). The World Health Organization (WHO) guidelines suggest that governments and public institutions, regardless of their political situation, clearly communicate information to the public in times of epidemic outbreaks (5, 6). During the 2003 Severe Acute Respiratory Syndrome (SARS) outbreak in China, the public highly relied on the social media as a reliable source instead of government and public officials (7). In addition, the Chinese population distrusted the reliability of information constructed by their government due to the lack of transparency in disseminating information on the outbreak (7).

According to Kasperson et al., many factors affect individuals' perception of risk, such as scientific information, interpersonal communication, cultural beliefs, and social interactions (8). An individual's response to a particular risk shapes their public perception of that risk itself. Risk is defined as “the product of the probability of an event occurring times the magnitude of potential consequences or impacts of that event” (9). The previous study documented that social media played a crucial role in forming an individual's risk perception of Middle East Respiratory Syndrome (MERS) because it was a useful tool in obtaining infectious disease information during an outbreak (10). It was documented by Balkhay et al. that the Saudi public had shown mistrust in the announcements of the Swine Flu outbreak by the Ministry of Health in the past (11). However, the public believed that not all cases could be detected (11). During outbreaks of infectious diseases, governments and public health officials inform the public about the situation and its risks (11, 12). They would use different media outlets such as television, newspapers, and social media to impact the publics' risk perception of these diseases and not merely to influence preventive behaviors and cognitive thought processes (12). Song et al. showed that people tend to positively share factual information and preventive measures regarding diseases on social media (13). Nevertheless, they can also express negative emotions and information resulting from their inner anxieties and fears. Intensive use of social media promotes shaping the public's risk perceptions of infectious diseases (13).

The current pandemic gives us a great opportunity to explore the factors that build public behavior and their adaptation of risk reduction measures (14). The risk perception and individual's subsequent behavior are complex and affected by multiple psychological and cultural factors (15). There are also rising concerns about COVID-19, information communicated on social media, and its impact on risk perception. This study builds on the previous study by Choi et al. in South Korea to yield additional insights into understanding the impact of social media on risk perceptions during an infectious outbreak (10). This study aims to determine the role of social media and its impact on the risk perceptions of the COVID-19 among the general population in Saudi Arabia. Understanding how social media relates to COVID-19 risk perceptions will help to facilitate future effective risk communications strategies, especially when faced with emerging public health threats.

## Materials and Methods

This was a cross-sectional study, and participants were recruited using different social media outlets between August to October 2020. This was the peak period of the pandemic. The survey was delivered through the Qualtrics platform and was delivered in English and Arabic. A convenience sampling technique was used to recruit participants to the study. An unrestricted self-selected survey link was posted on several social media platforms (WhatsApp, Facebook, and Twitter), and in response, 2,687 participants answered the survey. An unrestricted, self-selected survey is usually open to everyone to participate (16). Only Saudi Arabian residents who are over the age of 18 years old were able to participate in answering the survey. The face validity of the questionnaire in this study was determined by three faculty members within a similar discipline. The pilot respondents offered valuable feedback on the content of the questionnaire, and accordingly, unclear questions were modified. The questionnaire consisted of demographic information, aspects of risk perception, social media, and self-efficacy. The questionnaire was adapted from the previously validated and published study (10). A detailed description of each component is included in the following sections.

### Measurement

#### Risk perception

Risk perception was created using a Five-point, Likert scale with 1 as extremely unlikely and 5 as extremely likely. Two questions were asked to evaluate how much they agreed with the following questions; how likely do you think it is that you will be directly and personally affected by coronavirus/COVID-19 and how likely do you think it is that your friends and family will be directly affected by COVID-19. Mean values were created for these two questions in order to construct the index for risk perception. Mean = 3.55, *SD*: 0.82.

#### Social media

Social media exposure was assessed by inquiring about how often over the previous months they were exposed to news and information about COVID19 on social media, such as Facebook, Twitter, and WhatsApp. This was measured on a Five-point Likert scale with 1 as never and 5 as very often. Mean = 4.42, *SD*: 0.73.

#### Self-efficacy

Self-efficacy was measured by using 10 questions on a Five-point Likert scale with 1 as almost never and 5 as almost always. Participants were asked about mandatory standard operating protocols to avoid contracting COVID-19. Mean values were created by using these 10 questions to construct the self-efficacy index. Mean = 4.15, *SD*: 0.56.

#### Confounders

Certain sociodemographic variables were used as confounder variables. Age was categorized into the following categories: <20 years, 20–29 years, 30–39 years, 40–49 years, 50–59 years, and 60 years and above. Gender (male, female) and marital status (single, ever married) were categorized into binary variables. Education was categorized into high school and less, bachelor and individuals with graduate. Employment status was categorized into employed, unemployed, self-employed, retired, and student; and lastly income was categorized into individuals earning <4,000 Saudi Riyal (SR), 4,000 to <8,000 SR, 8,000 to <12,000 SR, and ≥12,000 SR.

### Statistical analysis

Hierarchical linear regression was used to explore the research hypothesis. Followed by socio-demographics, social media variables, self-efficacy, and attitude variables were entered into the simultaneous blocks. Dummy variables were created for age, employment, education, and income categories. An interaction term between social media and self-efficacy and social media and attitude were also created by using the standardized main effect variables. This was done to avoid multicollinearity between the interaction terms and their parent parts.

## Results

Table 1 demonstrates the sociodemographic characteristics of study participants. The mean age of participants was 32.5 (*SD*: 10.09), more than three quarters were of age 20–39 (72.1%), more than half were women (52.3%), less than one third were earning 12,000 and more Saudi Riyal (22.9%), were married (53.7%). Nearly half were employed or self-employed (47.5%) and more than half had bachelor's degree (59.4%).

**Table 1:** Sociodemographic characteristics of study participants (*N* = 2,680).

**Sociodemographic**	**Frequency (%)**
**Age**	
<20 years 20–29 years 30–39 years 40–49 years 50–59 years 60 years	166(6.2 ) 1,005(37.4) 937(34.9) 371(13.8) 145(5.4) 55(2.0)
**Gender**	
Male Female	1,277(47.6) 1,403(52.3)
Income*	
<4,000 4,000- <8,000 8,000- <12,000 >/=12,000	920(34.3) 334(12.4) 388(14.5) 615(22.9)
**Marital status**	
Single Married	1,241(46.3) 1,439(53.7)
**Education**	
High school and less Bachelor's Graduate and postgraduate	697(26.0) 1,595(59.4) 388(14.5)
**Employment**	
Unemployed Employed Self-employed Retired Student	611(22.8) 1,192(44.4) 77(3.1) 93(3.5) 707(26.3)

* *missing data*.

Mean risk perception was 3.55 (*SD*: 0.82), mean social media exposure was 4.42 (*SD*: 0.73), and mean self-efficacy score was 4.15 (*SD*: 0.56). In the first block of hierarchical linear regression, gender, monthly income, and marital status had a significant relationship with risk perception. Male gender, individuals earning 4,000–8,000, 8,000–12,000, and above 12,000, employed individuals compared to the female gender and those who are earning <4,000 Saudi Riyal had positive and significant relationship with the risk perception (β: 0.044, *p*-value: 0.035 and β: 0.051, *p*-value:0.041, β: 0.108 *p*-value: < 0.001, β: 0.119 *p*-value: < 0.001), respectively. While single individuals had a negative relationship with risk perception (β: −0.049, *p*-value: 0.043).

In the second block, social media was positively associated with the risk perception indicating that individuals exposed to social media had higher risk perception (β: 0.096, *p*-value < 0.001). In the third block, self-efficacy was significantly but negatively associated with the risk perception, indicating that individuals who were self-efficient were less likely to perceive the risk for COVID-19 (β: −0.096, *p*-value < 0.001). We could not find any interactive effect of social media and self-efficacy on risk perception (Table 2).

**Table 2:** Hierarchical linear regression model predicting risk perception toward Coronavirus disease 2019 (COVID-19) (*N* = 2,680).

**Block 1**	**risk perception**
**Age**	
<20 years(Ref)	
20–29 years 30–39 years 40–49 years 50–59 years 60 years	0.009 0.007 −0.005 0.007 −0.021
**Gender (1** **=** **male, 0** **=** **female)**	0.044*
**Education**	
High school and less (Ref)	
Bachelor's Graduate and postgraduate	−0.017 −0.001
**Income**	
<4,000 (Ref)	
4,000- <8,000 8,000- <12,000 >/=12,000	0.051* 0.108*** 0.119***
**Employment**	
Unemployed (Ref)	
Employed Self-employed Retired Student	−0.006 −0.042 −0.039 −0.021
Marital status (1 = single, 0 = married)	−0.049**
Incremental *R*^2^ (%)	3.3%
**Block 2**	
Social media	0.096***
Incremental *R*^2^ (%)	0.9%
**Block 3**	
Self-efficacy for COVID-19	−0.096***
Incremental *R*^2^ (%)	0.9%
**Block 4**	
Social media* Self-efficacy for COVID-19	0.005

* *p-value < 0.05*,

** *p-value < 0.01*,

*** *p-value < 0.001*.

## Discussion

The current study investigates demographic factors and social media's influence on the Saudi public's risk perceptions during the COVID-19 pandemic. The study aimed to understand the risk perceptions as it is critical for virus prevention and control of infections. The survey results confirmed that the risk perception is associated with various sociodemographic factors and social media. The study reported a key finding of higher risk perceptions among individuals with higher exposure to social media. It is in line with the previous research that documented that social media exposure was positively related to forming higher risk perceptions of South Korean individuals during the MERS outbreak (10). It has been previously suggested that people obtain information from media outlets during outbreaks, which impacts their risk perceptions of infectious diseases (17) and significantly improves preventive behaviors through self-relevant emotions (fear and anger). However, social media exposure to COVID-19 information also is associated with anxiety (18). A study reported that the use of social media is linked to the risk perception and self-efficacy, which in turn is associated with the preventive behavior during pandemics (19).

While the COVID-19 pandemic was looming, public health officials in Saudi Arabia selected optimal means of communication channels to disseminate information on the COVID-19 virus. The Ministry of Health and other government agencies are very active in using different social media platforms to make people aware of the virus and enforce protective behaviors. Such activities could have influenced the high-risk perceptions among the Saudi population, given that there was a high social media dependency of the public during the COVID-19 outbreak. The risk perception significantly impacts trust in the government and self-efficacy; this association was effective and helpful in avoiding virus exposure when social media was used for COVID-19 information (20).

The WHO recommended that communication outbreaks focus on gaining public trust and confidence, and governments must have transparency in disseminating information (6). Sociodemographic factors, such as male gender, individuals earning 4,000–8,000, 8,000–12,000, and above 12,000, employed individuals compared to the female gender, and those earning <4,000 Saudi Riyal had a positive and significant relationship with the risk perception. However, these results are consistent with a recent risk perception study in Iran; they found that men, being well-educated and married, believed they were more susceptible to the risks related to COVID-19 (21). In contrast, a large survey of individuals from ten countries indicates that men showed lesser risk perception (22). In particular, women in this study appeared to have lesser risk perceptions than men. Higher risk perceptions among men could be linked to the recent epidemiological data showing a greater incidence rate and severity of COVID-19 among men than women (23). One possible explanation is that the men may believe they are more susceptible to being infected. This is probably due to Saudi's cultural context that men occupy blue-collar jobs. Hence, they are more exposed and susceptible to the virus when on the job. On the contrary, these results are different from recent studies that indicated women had higher health risk perceptions of being infected with COVID-19 than men (24, 25). Women were more fearful about the debilitating consequences of COVID-19 than men, whereas men showed more aggressive behavior and self-efficient toward the COVID-19 pandemic (26). The previous studies have also documented that women perceived themselves as more susceptible to environmental risks than men (3).

The present study shows that participants with high education and income, being employed and married, had higher levels of perceived risk perceptions. Additionally, recent data also show that the higher education status was significantly associated with 72% lesser perceived high-risk against COVID-19. Several studies also reported similar findings that highly educated individuals carry out more protective and preventive behaviors against different pandemics, which is highly associated with other factors, such as attitudes and influences on their risk perceptions (27–29). Also, married individuals are more protective and worry about their families being infected. Meanwhile, it can be observed that employees whose working have a high-risk perception than those unemployed as they have more exposure when on the job.

The present study found that most respondents used social media very often. The International Telecommunication Union has noted that Saudi Arabia is one of the top ten countries using information and communication technology (30). There have been significant increases in social media users in Saudi Arabia over the past few years, reaching 23 million users, an amount equal to 70% of the population (31). Facebook is the most popular social media platform in the Kingdom, with over 15 million active monthly users. Instagram came in second with 12 million active monthly users. Additionally, Twitter had 11.27 million active monthly users as of January 2019 (31). According to the Ministry of Communications and Information technology in Saudi Arabia, approximately 18.3 million of the population use social media networks (Ministry of Communication and Information Technology, 2019).

During the COVID-19 pandemic and lockdowns, it not only helps in disseminating news but also information related to the virus, personal experiences, and perspectives with each other instantaneously (32). However, according to WHO, the current pandemic is also an “Infodemic,” where there is abundant information broadcasted about COVID-19 (33). Similarly, a study reported that frequent online information or social media usage leads to cyberchondria, information overload, or overconcern among individuals (34) timely and reliable dissemination of disease-related information. Developing countries usually have limited surveillance systems and resources to timely monitor infectious diseases outbreaks. Therefore, the social media act as a health networking mechanism to prevent the spread of disease in the community (19).

To date, there are no studies on the risk perception and its related factors to COVID-19 in Saudi Arabia, particularly the impact of social media on risk perceptions of a health pandemic. This study was carried out in Saudi Arabia during the highest recorded of COVID-19 patients; very high attention was given to the outbreak in the country. The results of risk perceptions and perceived susceptibility may differ with the decline in numbers and the less attention given to COVID-19 in the media. The study's major strength is that the survey was conducted in an ongoing pandemic. The study contributes to a better understanding of people's behavior during outbreaks and pandemics. The study also includes participants from various demographic categories. There are several limitations of the present study, which are worth to be mentioned. First, the study was conducted online, and all the data were based on self-reported measures, which could be under- or over-reported. Second, this was a cross-sectional study and only captured the snap short; therefore, we cannot comment on the causal relationship between the variables. A longitudinal prospective approach should be considered in future studies to make a stronger causal association. Third, this study used only a single variable to measure social media exposure. Although a single item cannot determine and cover the complete social media impact, as many individuals nowadays use various social media platforms. These platforms could have different influences, allowing users to create and distribute information. Therefore, it is worthy of examining the impact of all social media platforms separately and their influence on the risk perception. The results cannot be generalized because an unrestricted self-selected survey that is a form of convenience sampling, was used for this study.

## Conclusion

The current study results show that social media exposure to COVID-19 information has a positive impact on shaping an individuals' risk perception. Especially during lockdown, with easy accessibility of social media increases the dissemination of information about health issues. The study also suggests that there is a need for public officials and policymakers to develop effective communication strategies through risk communication campaigns targeted at women, individuals with lower socioeconomic status, and those who are single as they showed a negative relationship with the risk perception. It is essential that every country communicates with the general public about the disease's risks and provides them with basic information on disease transmission, management, high-risk practices, and protective behavioral measures during pandemics (35). Communication dramatically improves the general public's awareness and reduces the transmission of pandemic diseases in the past (35). It is suggested that disseminating clear information on disease transmissions and protective preventive measures to the public can significantly reduce transmissions (35).

During the outbreaks and pandemics, social media usage for the effective communication of risk susceptibility is important for public health and safety (36). Likewise, every individual's responsibility is to take personal precautions to decrease the spread of COVID-19 and understand that their own's decision has a huge impact on other individuals (37). An extension of this study should be considered to further identify factors and the impact of social media on risk perception and status of self-efficacy in a post-vaccination state. There might be a change of perception after the restrictions are lifted, but new variants are still emerging from different countries.

## Data Availability Statement

The original contributions presented in the study are included in the article/supplementary material, further inquiries can be directed to the corresponding author/s.

## Ethics Statement

The studies involving human participants were reviewed and approved by IRB of the Jeddah Research Health Affairs for science and technology. The participants provided their written informed consent to participate in this study.

## Author contributions

All authors listed have made a substantial, direct, and intellectual contribution to the work and approved it for publication.

## Conflict of interest

The authors declare that the research was conducted in the absence of any commercial or financial relationships that could be construed as a potential conflict of interest.

## Publisher's note

All claims expressed in this article are solely those of the authors and do not necessarily represent those of their affiliated organizations, or those of the publisher, the editors and the reviewers. Any product that may be evaluated in this article, or claim that may be made by its manufacturer, is not guaranteed or endorsed by the publisher.
